# Brassinazole Resistant 1 Activity Is Organ-Specific and Genotype-Dependent in Barley Seedlings

**DOI:** 10.3390/ijms222413572

**Published:** 2021-12-17

**Authors:** Jolanta Groszyk, Magdalena Szechyńska-Hebda

**Affiliations:** Plant Breeding and Acclimatization Institute—National Research Institute, 05-870 Błonie, Poland; m.szechynska@ihar.edu.pl

**Keywords:** 24-epibrassinolide, bikinin, brassinazole, BZR1, glycogen synthase kinase 3, GSK3, golden promise, Haruna Nijo, root, shoot

## Abstract

Brassinosteroids (BRs) control many plant developmental processes by regulating different groups of transcription factors, and consequently gene expressions. The most known is BZR1, the main member of the BES1 family. However, to date, it is poorly characterized in crop species. The main goal of the presented study was to identify *Hv*BZR1 and determine its activity in 5-day-old barley (the stage is related to one leaf on the main shoot and a few seminal roots) using two cultivars with different sensitivities to BRs. Using the anti-*Os*BZR1 antibody, we identified the forms of *Hv*BZR1 transcription factor with different molecular weights, which can be related to different phosphorylated forms of serine/threonine residues. Two phosphorylated forms in the shoots and one dephosphorylated form in the roots were determined. A minor amount of the dephosphorylated form of the *Hv*BZR1 in the Haruna Nijo shoots was also found. The phosphorylated forms gave a higher band intensity for Golden Promise than Haruna Nijo. The bands were similar in their intensity, when two different phosphorylated forms were compared in Golden Promise, while a reduced intensity was detected for the phosphorylated form with a lower molecular weight for Haruna Nijo. Degradation of the phosphorylated forms in the shoots (complete degradation in Golden Promise and significant but not complete in Haruna Nijo) and the presence of the dephosphorylated form in the roots were proven for the etiolated barley. In the case of Haruna Nijo, a wider range of the regulators of the BR biosynthesis and signaling pathways induced the expected effects, 24-EBL (0.001 µM) and bikinin (10 and 50 µM) caused low amount of the phosphorylated forms, and at the same time, a tiny band of dephosphorylated form was detected. However, the expression of genes related to the BR biosynthesis and signaling pathways was not a determinant for the protein amount.

## 1. Introduction

Brassinosteroids (BRs) are a class of plant polyhydroxylated steroid hormones [1,2] that control plant growth and architecture, including leaf morphology and lamina joint inclination [3,4,5], photomorphogenesis [6], and grain size, by the regulation of many genes and transcription factors (TF) [4,7,8,9,10,11,12]. Recently, BR biosynthesis and signaling pathways were determined for different crop species, e.g., experimentally in rice (*Oryza sativa* L.), common wheat (*Triticum aestivum* L.), maize (*Zea mays* L.), brachypodium (*Brachypodium distachyon* L.), and barley (*Hordeum vulgare* L.) [12,13,14,15,16,17,18]. Moreover, the results were reviewed in a few papers [15,19,20,21,22].

In arabidopsis (*Arabidopsis thaliana* L.), the last step of the BR signaling pathway leads to the regulation of Brassinazole Resistant 1 (BZR1) activity. The BZR1 is a DNA-binding protein, and regulates thousands of the BR-responsive genes [23,24,25]. A reduced transcript level of *OsBZR1* led to the dwarf rice phenotype, its erected leaves, and reduced sensitivity to BRs [26]. *TaBZR2* overexpression improved the drought tolerance (survival rate) of wheat as a reduced electrolyte leakage and malondialdehyde content. The higher proline content was observed during drought conditions [16].

The BZR1 with other TFs are regulated by the Brassinosteroid Insensitive 2 (BIN2) kinase from the Glycogen Synthase Kinases 3 (GSK3s) family. *Os*GSK2, an ortholog of BIN2 in rice, is also one of the critical suppressors of the BR signaling pathways. Therefore, it plays an important role in plant architecture [5,27,28,29,30,31]. Both *Os*GSK2 and *Os*BZR1 are involved in the regulation of the Dwarf and Low-Tillering (*Os*DLT) [32], Leaf and Tiller Angle Increased Controller (*Os*LIC) [33], Reduced Leaf Angle 1/Small Organ Size 1 (RLA1/SMOS1) [34], Growth-Regulation Factor 4 (*Os*GRF4) [7], Ovate Family Protein 1 (*Os*OFP1) [29], and Ovate Family Protein 3 (*Os*OFP3) [5]. Moreover, the BZR1, directly bound to the promoter of various genes, regulates the signal transduction and biosynthesis of different hormones: gibberellins, abscisic acid, auxins, ethylene, cytokinins, and jasmonates [35,36,37,38,39]. The *Os*BZR1 transcriptional activity is inhibited by its cytoplasmic localization [26], which in turn, is controlled through serine phosphorylation by *Os*GSK2 [40,41].

Genes that control consecutive stages of the BR signaling pathway were identified in barley. Two genes, i.e., *HvBRI1* [42] and *HvBAK1* [43], encode the Brassinosteroids Insensitive 1 (BRI1)—BRI1 Associated Receptor Kinase 1 (BAK1) transmembrane receptor. The BRI1-BAK1 captures signaling molecules with a brassinolide (BL, the final product of the BR biosynthesis pathway) structure leading to a change in the protein conformation and activation of the BR signaling pathway. The *HvBSU1* encodes the phosphatase BSU1, which regulates the activity of proteins from the GSK3s family [40]. In barley, the GSK3s family is represented by seven genes, all of which have the orthologues in arabidopsis [44] and rice [45]. The genes, different in their sequence and functions, were assigned to four groups [46]. The first group consists of *HvGSK1.1*, *HvGSK1.2*, and *HvGSK1.3*; the second group consists of *HvGSK2.1* (an orthologue of *AtBIN2* and *OsGSK2*) and *HvGSK2.2*; the third group consists of *HvGSK3.1*; and the fourth group consists of *HvGSK4.1.* The gene encoding *Hv*BZR1 was not earlier investigated in barley.

The main goal of the presented study was to identify the *Hv*BZR1 and determine its activity in 5-day-old barley shoots and roots (the stage is related to one leaf on the main shoot and a few seminal roots). The 24-Epibrassinolide (24-EBL), BIN2 Kinase Inhibitor (bikinin), and Brassinazole (Brz) were used to regulate the BR biosynthesis and signaling pathways experimentally [18]. The 24-EBL, the most active phytohormone, activates the BR signaling pathway and leads to the inhibition of GSK3s [47]. Bikinin activates BR signaling downstream of the BR receptor, directly binds the BIN2, and acts as an ATP competitor. Moreover, bikinin inhibits the activity of six other arabidopsis GSK3s [48]. In contrast, Brz is a specific BR biosynthesis inhibitor. A target site of Brz is the conversion of campestanol [49]. Brz inhibits the enzymatic activity of DWF4, and as a consequence, promotes the activity of GSK3s, and limits BR signaling pathways [49]. Our study was designed to distinguish the phosphorylated and dephosphorylated forms of the *Hv*BZR1. Here, we assumed that the treatment with 24-EBL or bikinin allows for the identification of the dephosphorylated form of the *Hv*BZR1 at higher concentrations, while Brz allows for the identification of the phosphorylated form of the *Hv*BZR1. To date, the occurrence of both BZR1 forms was only verified by gene expression in rice protoplast or tobacco leaves [50,51], and in protein extract from rice anthers [52] or leaves [51,53].

## 2. Results

### 2.1. HvBZR1 Activity, Plant Phenotype, and BR-Related Gene Expression

**Figure 1:**
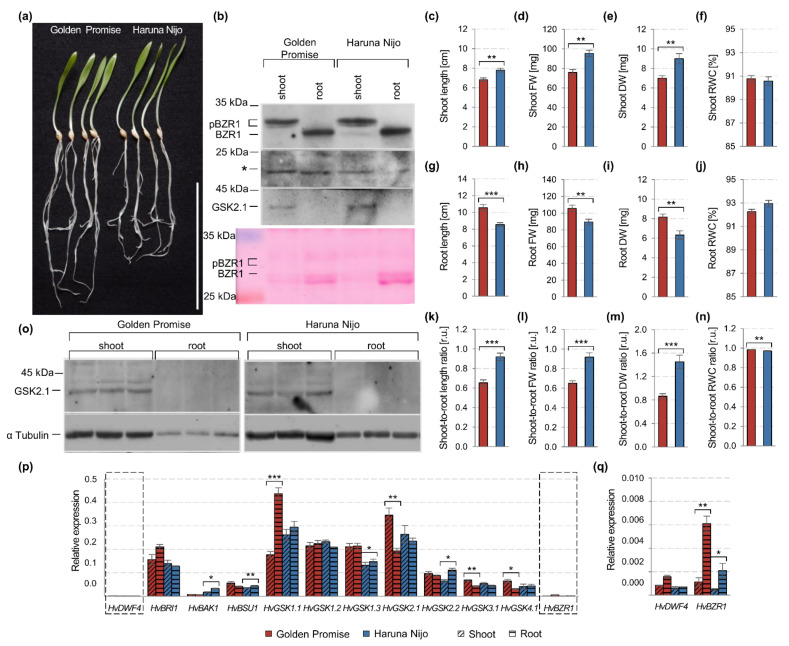
Phenotypic and molecular traits of 5-day-old shoots and roots measured for the Golden Promise and Haruna Nijo cultivars. (**a**) Representative example of plant phenotype in four biological replicates, scale bar = 10 cm; (**b**) immunodetection of *Hv*BZR1 and *Hv*GSK2.1, as well as a membrane staining of protein bands with a molecular weight in range from 25 to 35 kDa using the Ponceau S; * indicates a nonspecific product detected by the anti-*Os*BZR1 antibody; pBZR1 indicates the phosphorylated *Hv*BZR1 forms; BZR1 indicates the dephosphorylated *Hv*BZR1 form; (**c**–**n**) parameters of shoot growth (**c**–**f**), root growth (**g**–**j**), and shoot-to-root ratio (**k**–**n**); (**o**) immunodetection of *Hv*GSK2.1 kinase and α-tubulin as a control; (**p**,**q**) expression profile of the genes related to the BR biosynthesis and signaling pathways. The results present the mean with a standard error of the mean (phenotypic traits *n* = 6, (**c**–**n**); expression profile *n* = 3, (**p**,**q**). The asterisks indicate significant differences, revealed by the Student’s *t*-test for *p* < 0.05 (*), *p* < 0.01 (**), and *p* < 0.001 (***).

A sequence identity search with the Basic Local Alignment Search Tool (BLAST) program in the UniProt Knowledgebase [54] for *Os*BZR1 (Q7XI96) [26,30,31,33,50] was performed. The F2CRW8 of *Hordeum vulgare* subsp. *vulgare* with an 83.6% identity of the sequence was found (Figure S1). The bioinformatic analysis showed a high degree of identity between *Os*BZR1 and putative BZR1 orthologs in the other crop species, i.e., common wheat, 86.3–85.6%; foxtail millet (*Setaria italica* L.), 82.6%; broomcorn millet (*Panicum miliaceum* L.), 82.8%; sorghum (*Sorghum bicolor* L.), 78.7%; and brachypodium, 76.9%. The F2CRW8 consists of 317 amino acid residues (aa) and has BZR1 typical domains, i.e., DNA binding domain, 14-3-3 domain, PEST domain, and EAR motif (Figure S1). The molecular weight (MW) of F2CRW8 protein is 33.9 kDa, and the isoelectric point (IP) is 8.0537; while the MW of *Os*BZR1 (298 aa) is 31.9 kDa, and IP is 8.4966 (Ensemble Plants). The F2CRW8 protein sequence is deposited with an accession number BAJ85589.1, and a coding sequence with accession number AK354370 in the National Center for Biotechnology Information (NCBI) or HORVU.MOREX.r2.2HG0108340 Ensemble Plants databases [55]. Using the BLASTN program, the nucleotide database was searched for the highly similar sequences (megablast) and the results indicated an 86.9% identity between *H. vulgare* subsp. *vulgare* AK354370 sequence and rice Os07t0580500-01 (Ensemble Plants, *O. sativa* Japonica Group, IGRSP-1.0 [56,57], older version Os07g39220.1 [26,34]), encoding *OsBZR1* [26,30,31,33,50] (Figure S2). The AK354370 sequence was used for the construction of the expression vector and F2CRW8 recombinant protein. The F2CRW8 recombinant protein was detected at the MW of ~30 kDa (Figure S3). This band corresponds to the dephosphorylated form of *Os*BZR1 [51,58,59] and *Si*BZR1 [60]. The Western blot analysis performed for the proteins extracted from shoots and roots of 5-day-old barley seedlings (Figure 1a) allowed for the identification of the presence of *Hv*BZR1 in three different forms (Figure 1b). Two bands, thick one and thin one, located very close to each other, were obtained after analysis of the protein extract from the shoots of both cultivars, Golden Promise and Haruna Nijo. Another form represented by a thick band with a lower MW was found in the roots of both cultivars, and in a low amount in Haruna Nijo shoots. Small differences in the MW of the *Hv*BZR1 phosphorylated forms can result from a diverse level of phosphorylation on serine and threonine residues, which in turn, is a major mechanism regulating the activity of cell proteins and playing a central role in signal transduction pathways [26,50,61]. The number of phosphorylation sites of serine and threonine residues is dependent on the species; there are 28 phosphorylation sites of serine and threonine residues in barley and rice (Figure S1), and 21 in arabidopsis [26].

**Figure 2:**
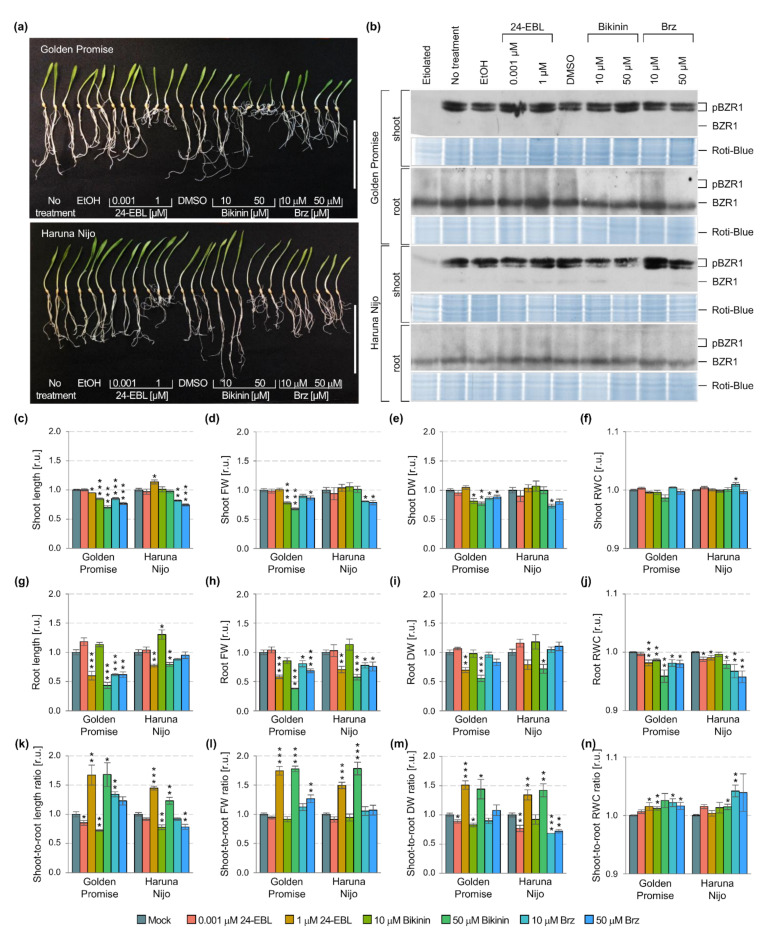
Phenotypic and molecular changes in 5-day-old shoots and roots of the Golden Promise and Haruna Nijo plants treated with 24-EBL (0.001 and 1 µM), bikinin (10 and 50 µM), and Brz (10 and 50 µM). EtOH (0.7%) was used as a control of solvent solutions for 24-EBL, while DMSO (0.7%) was the control of solvent solutions for BK and Brz. Mock represents the plants treated with the respective solvent solutions, i.e., EtOH and DMSO assumed as 1.00. (**a**) A phenotype of plant photography represents three biological replicates for each treatment, scale bar = 10 cm; (**b**) immunodetection of *Hv*BZR1, the Roti-Blue presents proteins after staining as a loading control; pBZR1 indicates the phosphorylated forms of the *Hv*BZR1; BZR1 indicates the dephosphorylated form of the *Hv*BZR1; (**c**–**f**) growth parameters of shoots, (**g**–**j**) roots, and (**k**–**n**) shoot-to-root ratio. The results present the mean with a standard error (*n* = 6). The asterisks indicate significant differences, revealed by the Student’s *t*-test for *p* < 0.05 (*), *p* < 0.01 (**), and *p* < 0.001 (***).

A few works were focused on the detection of the *Os*BZR1 protein in rice, while the *Hv*BZR1 has not been previously studied in barley at all. Inactivation of the phosphorylated forms of the BZR1 by Constitutive Photomorphogenic 1 (COP1) in dark conditions and degradation of the dephosphorylated form of the BZR1 by SINA E3 ubiquitin ligases in the light were previously proven [20,62,63]. Therefore, we compared plants grown in the light with those grown in dark conditions (5-day-old etiolated plants) to prove F2CRW8 as *Hv*BZR1 (Figure 2b). For the first time, degradation of the phosphorylated forms of the *Hv*BZR1 in the shoots (complete degradation in Golden Promise, and significant, but not completed degradation in Haruna Nijo), and the presence of the dephosphorylated form of the *Hv*BZR1 in the roots were proven for etiolated barley seedlings (Figure 2b). The results indicated, that the *Hv*BZR1 activity was organ-specific. Similarly, the identification of phosphorylated *Hv*BZR1 forms corresponded to the *Hv*GSK2.1 appearance in barley shoots (Figure 1b,o), while the root-specific dephosphorylated *Hv*BZR1 form was related to *Hv*GSK2.1 absence. This can be considered as a specific feature for *Poaceae* or crop species.

To link the influence of *Hv*BZR1 activity with barley phenotype, the morphology of 5-day-old plants was assessed. All of the parameters describing the growth of shoots and roots showed a similar cultivar-dependent pattern (Figure 1a,c–n). The shoots of the Haruna Nijo were longer, and their fresh weight and dry weight were improved, when compared to the Golden Promise (Figure 1c–e). The opposite effect was found for the roots; their length and weight were significantly higher in the Golden Promise (Figure 1g–i). In all cases, the relative water content was similar (Figure 1f,j). The result indicates that despite the reduced root growth, which may directly affect the efficiency of water and nutrient uptake, the Haruna Nijo was able to develop much stronger shoots (Figure 1k–n). These traits differentiated both genotypes and suggested that growth changes could be related to the difference in the dephosphorylated *Hv*BZR1 form in the shoots of both cultivars (a small amount in the Haruna Nijo, and its absence in the Golden Promise, Figure 1b).

The BZR1 activity is regulated by the BIN2 as well as other GSKs in the BR signaling pathway [26]. Therefore, we determined the expression of key genes in this pathway (Figure 1p,q). The BL molecules are used to initiate the BR signaling pathway. Therefore, we also determined the expression of the first gene controlling the BR biosynthesis, i.e., *HvDWF4*. The *DWF4* expression is regulated by BZR1, and the DWF4 activity is inhibited by Brz [64]. *HvBRI1* and *HvBAK1* encode a transmembrane receptor BRI1-BAK1, which captures the BR molecules with the BL structure (i.e., 24-EBL). *HvBSU1* encodes the phosphatase BSU1, which is regulated by the BR signaling pathway and inhibits proteins from the GSK3s family. Moreover, we analyzed the expression of genes encoded GSK3s family members [46], and finally the *HvBZR1* expression. The genes encoding enzymes that control the subsequent steps of the BR signaling pathway were expressed in cultivar- and organ-dependent mode (Figure 1p,q). The Real-Time PCR analysis allowed to distinguish four types of gene responses. The first group consisted of the genes in which the expression was organ-dependent in the Golden Promise, but at the same time, they did not change in the shoots and roots of the Haruna Nijo. These were the genes specifically upregulated in the roots (*HvDWF4*, *HvBRI1*, *HvGSK1.1*) and in the shoots (*HvBSU1*, *HvGSK2.1*, *HvGSK3.1*, *HvGSK4.1*) of the Golden Promise. The second group consisted of genes upregulated in Haruna Nijo roots (*HvBAK1* and *HvGSK2.2*), while organ-independent in the Golden Promise. The third group included two genes with a similar expression in the organs of both cultivars (*HvGSK1.2* and *HvGSK1.3*). However, one of them (*HvGSK1.3)* was clearly cultivar-dependent, as a significantly higher expression was detected in both the shoots and roots of the Golden Promise. The *HvBZR1* represented the fourth type of response, as the upregulation in the roots, but not the shoots of both cultivars, was found. The result for *HvBZR1* was consistent with data for other plant species [61].

Despite the relatively low expression of *HvBZR1* (compared to the other genes, Figure 1p), the strong signal of the dephosphorylated *Hv*BZR1 form (Figure 1b) and the absence of *Hv*GSK2.1 (Figure 1b,o) suggest an important role for this TF in the regulation of barley root development. On the other hand, the presence of the dephosphorylated *Hv*BZR1 form (Figure 1b) in the Haruna Nijo shoots can be genotype-dependent and correlated with sensitivity to BRs. Our previous study, confirms this statement: A greater sensitivity of Haruna Nijo to 24-EBL, bikinin, and Brz treatment was determined by the lamina joint inclination test [18].

### 2.2. Barley Response after 24-EBL, Bikinin, and Brz Treatment

To determine the contribution of the phosphorylated and dephosphorylated forms of *Hv*BZR1 in the regulation of plant growth, the Golden Promise and Haruna Nijo cultivars were treated with 24-EBL, bikinin, and Brz at the two concentrations. This differentiate the shoot and root phenotypic traits between the cultivars in our previous study [18]. The 24-EBL and bikinin lead to the inhibition of GSK3s activity, and consequently promote the dephosphorylation of BZR1 and its nuclear localization [50,61]. In addition, 24-EBL inhibited the GSK3s activity through the activation of the BR signaling pathway, while bikinin through the inhibition of LEYV (specific for the second group of the GSK3s family members) and MEYV motif (specific for the first and third groups of GSK3s family members) [48]. On the contrary, the Brz treatment leads to an inhibition of the BR biosynthesis pathway and impaired BR signaling pathway, thus allowing for the GSK3s to be active, and consequently promotes the phosphorylation of BZR1 and its cytoplasmic localization. In each case, the concentration of the active substance was selected based on the earlier studies, which focused on their dose-dependent regulation of barley growth [18]. The 0.001 µM 24-EBL and 10 µM bikinin stimulated root elongation, while 1 µM 24-EBL, 50 µM bikinin, 10 µM Brz, and 50 µM Brz inhibited barley shoot and root development (Figure 2a,c–n).

In the case of each treatment (24-EBL, bikinin, and Brz), the bands typical for the untreated control plants were detected, i.e., two phosphorylated forms of the *Hv*BZR1 in a large amount in shoots of both cultivars, a major amount of dephosphorylated form in roots of both cultivars, and a minor amount of dephosphorylated form in shoots of the Haruna Nijo (Figure 2b). A more detailed analysis of bands showed differences in the amount of *Hv*BZR1 between the cultivars and after the treatment with active substances (Figure 2b). In shoots, phosphorylated forms had higher band intensities for Golden Promise than Haruna Nijo. The bands were similar in their intensity when two phosphorylated forms were compared in Golden Promise, while a reduced intensity was detected for the phosphorylated form with a lower MW for Haruna Nijo. Similarly, in roots, the dephosphorylated form with a higher content for Golden Promise than Haruna Nijo was found. The expected (similar as presented in the literature for other species) response in shoots was only observed after the treatment of Golden Promise plants with bikinin used in the low concentration (10 µM). A low amount of phosphorylated forms was noted, even if dephosphorylation was not observed (absence of bands related to the dephosphorylated form). In the case of Haruna Nijo, the wider range of treatments induced the expected effects. 24-EBL in a low concentration (0.001 µM) and bikinin at both concentrations (10 and 50 µM) caused the low amount of phosphorylated forms, and at the same time, the tiny band of dephosphorylated form was detected. In the same way, Brz at both concentrations (10 and 50 µM) induced an accumulation of phosphorylated forms and promoted the absence of dephosphorylated form in Haruna Nijo shoots. In Golden Promise roots, the expected response was found after the 1 µM 24-EBL treatment, i.e., a high level of dephosphorylated form. For the other treatments, the effects were relatively comparable to the untreated control, particularly in Haruna Nijo root samples. The shoot-to-root ratio of both cultivars increased after the treatment with 1 µM 24-EBL and 50 µM bikinin, but decreased when a low concentration of the active substances was used. Brz led to genotype-dependent changes in the shoot-to-root ratio, i.e., increased parameters in Golden Promise and decreased in Haruna Nijo. Altogether, the results proved the organ-specific (occurrence of phosphorylated forms of the *Hv*BZR1 in the shoots, and dephosphorylated form of the *Hv*BZR1 in the roots) and genotype-dependent (major changes in *Hv*BZR1 for Haruna Nijo shoots, minor changes in *Hv*BZR1 for Golden Promise shoots) activity of *Hv*BZR1.

Following the changes in *Hv*BZR1 activity after the different treatments, the plant growth changes induced by active substances were also organ-specific and genotype-dependent. In the first case, roots were more sensitive to different concentrations of active substances than shoots, particularly to 24-EBL and bikinin. The pattern of changes was similar in both cultivars, in which a low concentration increased the growth parameters and a high concentration decreased them. In the second case, shoot growth was more dependent on the genotype. The Golden Promise had decreased the shoot growth after the 24-EBL and bikinin treatment, in contrast to the improved growth of the shoots of Haruna Nijo.

**Figure 3:**
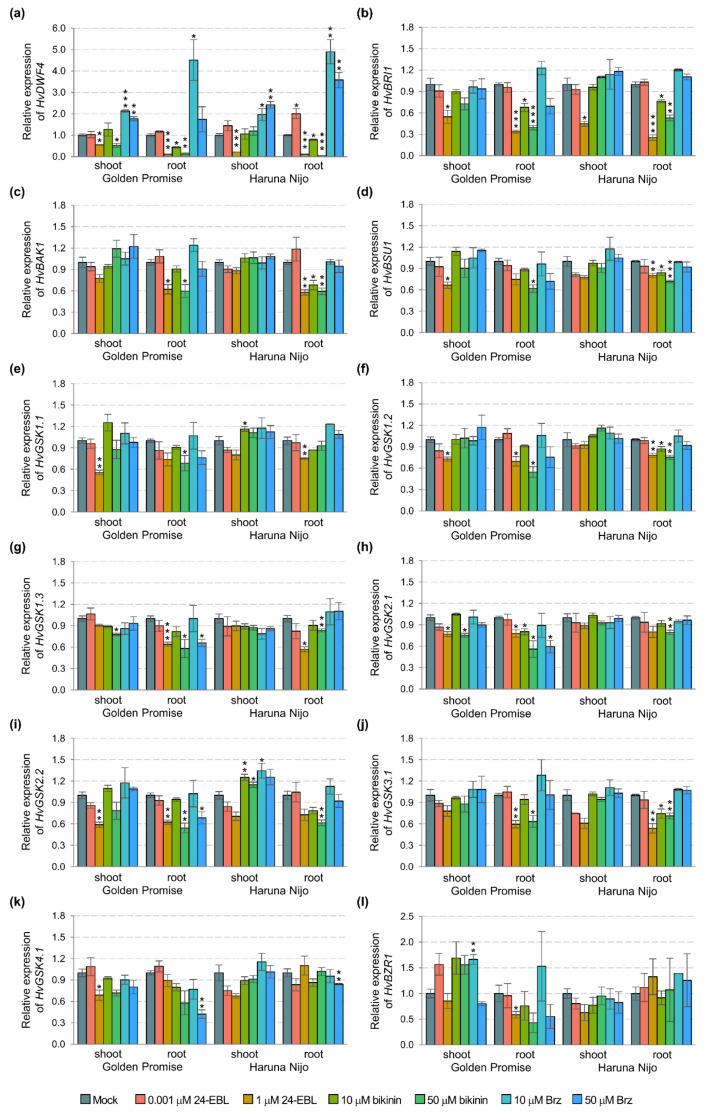
Expression profiles of the genes encoding enzymes of BR biosynthesis and signaling pathways in two barley cultivars Golden Promise and Haruna Nijo. Shoots and roots were treated with 24-EBL (0.001 and 1 µM), bikinin (10 and 50 µM), and Brz (10 and 50 µM), mock represents the plants treated with the respective solvent solutions, i.e., EtOH and DMSO assumed as 1.00. The results present the mean with a standard error (*n* = 3). The asterisks indicate significant differences, revealed by the Student’s *t*-test for *p* < 0.05 (*), *p* < 0.01 (**), and *p* < 0.001 (***).

In addition, a detailed statistical analysis proved that all of the measured traits in shoots and only dry weight in roots were genotype-dependent (Table 1). Following the results, we performed a quantitative analysis of the genes controlling consecutive stages of the BR signaling pathway (Figure 3). 24-EBL and bikinin at the high concentration reduced expression of the genes in barley roots, e.g., *HvBSU1*, *HvGSK2.1*, and *HvGSK2.2*. Similarly, the 10 µM bikinin resulted in a significant reduction of the expression of some genes in the roots of Haruna Nijo (*HvBAK1*, *HvBSU1*, and *HvGSK1.2*) and in Golden Promise (*HvGSK2.1*). In the shoots, the 50 µM 24-EBL reduced expression of the genes in Golden Promise, while most of the genes were not altered in Haruna Nijo. In Golden Promise, a few genes were downregulated by Brz, which was applied in the high concentration. Two-way ANOVA indicated that most of the genes (with the exception of the *HvBRI1*) in shoots and only *HvGSK1.1* and *HvGSK2.1* in the roots are genotype-dependent (Table 1). The results indicated that the following steps of the BR signaling pathway can be organ-specific and genotype-dependent in 5-day-old barley seedlings.

## 3. Discussion

In the last few years, the role of GSK3s and BZR1 was extensively studied in rice architecture and grain formation [12,51,60,65]. However, the proteins were rarely studied at the early developmental stages and during stress responses. Similarly, few articles have provided information regarding GSK3s in barley [18,46], and there is no available data concerning *Hv*BZR1. In our study, we present the organ-specific *Hv*BZR1 activity and genotype-dependent responses to the active substances, which modify the BRs biosynthesis and signaling pathways, i.e., 24-EBL, bikinin, and Brz.

The barley cultivars Golden Promise and Haruna Nijo displayed a different sensitivity to 24-EBL, bikinin, and Brz in the lamina joint inclination test [18]. They had different phenotypes, most probably as a result of differences in the BR-related pathways. Some works indicated that the lamina joint depends on the endogenous BRs level [51,60]. Our experiments proved that different phenotype of both cultivars corelate with different levels of *Hv*BZR1 and *Hv*GSK2.1 proteins. *Hv*GSK2.1 is a homolog of arabidopsis BIN2 and rice *Os*GSK2 protein kinases [46]. Here, the most important result consists of a different pattern of the *Hv*BZR1 forms between both cultivars. Similar to *Os*BZR1 that was detected in the protoplasts of wild-type rice plants [50], we detected two phosphorylated forms and one dephosphorylated form of *Hv*BZR1 in barley with different MW levels. Differences in MW correspond to various levels of phosphorylation in serine and threonine residues [26,51]. Furthermore, in the dark condition, phosphorylated forms of the *Hv*BZR1 degraded completely in the shoots of the Golden Promise, while a significant but not completed degradation was found in the Haruna Nijo.

The treatment of a 5-day-old barley plant with 24-EBL and bikinin was expected to promote the dephosphorylated *Hv*BZR1 form, and the Brz treatment to promote the phosphorylated *Hv*BZR1 forms. Unexpectedly, we found that the *Hv*BZR1 activity was strongly organ-dependent, and the *Hv*BZR1 divergent pattern was found between the shoot and root tissues (Figure 2b). The phosphorylated forms were detected in the shoots and the dephosphorylated form in the roots. Their amount was only slightly modified by the active substances. The result was strengthened by the *Hv*GSK2.1 analysis. *Hv*GSK2.1 was present in the shoots, but it was undetected in the roots of both barley cultivars. The activity of PUB40, the U-box E3 ubiquitin ligase, led to the degradation of both forms of the BZR1 in arabidopsis roots, but not in the shoots, when BRs were present in the low amount [61], and *Os*PUB24 induced *Os*BZR1 proteasomal degradation by ubiquitination of *Os*BZR1 in rice [50]. Both ligases required phosphorylation by *Os*GSK2/BIN2 to induce their activity. Earlier studies with protoplasts allowed for the study of *Os*BZR1 changes only in the shoots [50]. For the first time, our study proved the organ-specific location and response of *Hv*BZR1 in barley shoots and roots at the early stage of plant development.

A higher amount of the phosphorylated *Hv*BZR1 forms in shoots than roots and the difference between two phosphorylated forms in Golden Promise (an equal amount of both forms) and Haruna Nijo (a lower amount of the form with lower MW), may be related to the *Hv*BZR1 specific role in the regulation of plant aboveground development. In the case of Haruna Nijo, the wider range of treatments induced the expected effects, 24-EBL and bikinin caused the low amount of phosphorylated forms and at the same time, the tiny band of dephosphorylated form was detected. The pleiotropic effect was suggested in the case of bikinin [48]. Indeed, results consisting of the significant degradation of phosphorylated forms of the *Hv*BZR1 in Haruna Nijo, its stability in the Golden Promise plant, and GSK3s inhibition. Altogether, it raises the following questions: 1. Is the *Hv*BZR1 activity regulated by GSK3s?; 2. and/or is the regulation determined by the affinity of bikinin for the bikinin-binding domains, i.e., LEYV in GSK group II (GSK2.1 and GSK2.2), MEYV in group I (GSK1.1, GSK1.2, and GSK1.3), and III (GSK3.1) ? The hypothesis was partially supported by the results of gene expression. We observed a lower expression of the *HvBZR1* gene in the shoots than the roots of barley. Similarly, a higher expression of *BZR1* was observed in arabidopsis and rice roots [50,61]. However, the expression of genes related to BR biosynthesis and signaling pathways, in our case, was not a determinant for the protein amount. For instance, the upregulation of *HvGSK2.1* was not followed by the improved amount of *Hv*GSK2.1 protein in barley roots.

Recent studies with arabidopsis mutants related to individual steps of the BR biosynthesis pathway indicated a highly site-specific BRs function and short-distance signal transduction in roots [66]. The nuclear localization of BZR1 was specific for the root meristem zone, and its role was assigned to cell division, particularly the Quiescent Center (QC), Columella Cell (CC), and Columella Stem Cell (CSC) [67,68]. The QC of the root meristem is under control of the Brassinosteroids at the Vascular and Organizing Centre (BRAVO) TF, inhibiting the QC division [69]. In addition, 24-EBL or bikinin treatment can lead to GSK3 deactivation, thus the activation of BZR1, and inhibition of BRAVO. As a result, this can induce cell division and root elongation. Indeed, both barley cultivars had elongated roots after the treatment with low concentrations of 24-EBL and bikinin, and the elongation resulted from the cell division rather than from the cell elongation (improved fresh and dry weight, stable RWC). On the contrary, roots phenotype changes after the plant treatment with a high concentration of 24-EBL and bikinin were consistent with the GSK3 role in the regulation of auxin signaling pathway [68,70,71], and changes in the PIN-Formed 2 (PIN2) level [72,73]. The changes in Golden Promise and Haruna Nijo root phenotypes suggest that the application of 24-EBL and bikinin, but not Brz, led to a modification in auxin signaling, but the hypothesis should be verified in the future.

The presented results confirmed that effect of 24-EBL, bikinin, and Brz on barley development is dose- and genotype-dependent as well as organ- and developmental stage-specific (Figure 2) [74,75]. However, considering our results and the other studies, the important differences in both, the role of *Hv*BZR1 in plant growth and the effect of active substances in the modification of the BR biosynthesis and signaling pathways, were recognized when barley (monocotyledon) was compared to arabidopsis (dicotyledon). In conclusion, similar phenotypical changes after the bikinin and 24-EBL treatments suggest a role of GSK3s in the regulation of *Hv*BZR1 activity. *Hv*BZR1 can be localized in the nucleus of root cells, and its activity can lead to the BR-dependent regulation of gene expression. In barley shoots, *Hv*BZR1 can be localized mainly in the cytoplasm. The phosphorylation or dephosphorylation of serine and threonine allow for the interaction of *Hv*BZR1 with other proteins. Finally, phenotype changes are also dependent on the different plant sensitivity to BR, as we observed for Golden Promise and Haruna Nijo. However, further work is required to determine the factors influencing the plant sensitivity to BRs, and the role of dephosphorylated and phosphorylated forms of the BZR1.

## 4. Materials and Methods

### 4.1. Chemicals

The active substances: 24-epibrassinolide (24-EBL, CAS 78821-43-9, purity ≥85%), bikinin (CAS 188011-69-0, purity ≥98%,), and brassinazole (Brz, CAS 224047-41-0, purity ≥98%,) were purchased from Sigma-Aldrich (Schnelldorf, Germany). Dilution of 24-EBL was prepared from 1 mM 24-EBL stock dissolved in 70% ethanol (EtOH) (POCH, Gliwice, Poland). Dilution of bikinin and Brz was prepared from 91.5 mM and 76.3 mM stock, respectively, dissolved in 100% Dimethyl Sulfoxide (DMSO) (Sigma-Aldrich, Schnelldorf, Germany). Controls of the experiments constituted the same concentration of the solvent solution, which was used as a background for the dilution of each chemical.

### 4.2. Plant Material

Each barley (*Hordeum vulgare* L.) cultivar presents the single seed descent (SSD) line. Golden Promise (United States Department of Agriculture, GRAIN-Global, USA, accession number 343079) and Haruna Nijo (Gene Bank Dept., CRI Prague—Ruzyně, accession number 03C0602168) were used in the experiments. The grains grew in a single Petri dish with three layers of filter paper and 15 mL of spring water (Żywiec-Zdrój S.A., Węgierska Górka, Poland). They were kept at 4 °C in dark conditions for 72 h, then at 23 °C for 72 h. Next, six plants were planted in the pots (27 × 21 × 17 cm) filled with soil substrate (HolLas, Pasłęk, Poland) and sand (4:1). Plants grew in a growth chamber at 18/16 °C and 16-h photoperiod with the light intensity of about 200 µmol photons m^−2^ s^−1^, and humidity of 70%. Weekly, plants were supplemented with the Florovit (INCO—GRUPA S.A., Warszawa, Poland) solution. Grains from each single plant were collected separately and used for the experiments.

### 4.3. Treatments and Growth Traits

Grains were germinated in the presence of 0.001 and 1 µM 24-EBL, 10 and 50 µM bikinin, 10 and 50 µM Brz, 0.7% EtOH (control for 24-EBL), and 0.7% DMSO (control for bikinin and Brz). Plants were cultivated as previously reported [18]. The 5-day-old barley shoots and roots (the stage is related to one leaf on the main shoot and a few seminal roots) were collected between 10:00 and 12:00. Plants were photographed, and the shoot and root length were measured with the ImageJ software (v. 1.51k). The shoot and root length were measured between the mesocotyl and the 1st leaf tip and between the mesocotyl and the tip of the longest primary root, respectively. Plants were weighed, the fresh (FW) and dry weight (DW) were used to calculate the relative water content (RWC) according to the formula: RWC = (Fw − Dw/Fw) × 100%.

### 4.4. Western Blot Detection of GSK2.1 and BZR1 Proteins

Total proteins were extracted from the shoots and roots of the Golden Promise and Haruna Nijo. The plant material was ground into a fine powder in liquid nitrogen. The samples were treated with a 1 × SDS sample buffer (5 µL per 1 mg of shoots, 2 µL per 1 mg of roots) at 70 °C for 10 min, then centrifuged. The supernatant was used for the SDS-PAGE electrophoresis (Figure 1b, 3 h; Figure 2b, 4.5 h) and Western blot. Commercial polyclonal antibodies: Anti-*Os*GSK2, AbP80050-A-SE (Beijing Protein Innovation Co., Beijing, China), Brassinazole resistant 1 (*Oryza sativa*), AS16 3219 (Agrisera, Vännäs, Sweden), and tubulin alpha chain, AS10 680 (Agrisera, Vännäs, Sweden) were used to detect *Hv*GSK2.1, *Hv*BZR1, and tubulin, respectively. Secondary antibodies: Goat anti-rabbit IgG (H&L), HRP conjugated, and AS09 602 (Agrisera, Vännäs, Sweden) were used with dilution 1:100,000. Luminescent detection was performed with the AgriseraECL SuperBright, AS16 ECL-S (Agrisera, Vännäs, Sweden) and Ilford Delta 3200 (Harman Technologies, Cheshire, UK).

### 4.5. RNA Extraction, cDNA Synthesis, and Real-Time PCR Analysis

Total RNA was extracted using the TRI Reagent Solution (Life Sciences Solutions, Carlsbad, CA, USA). The cDNA was synthesized using the Maxima H Minus First Strand cDNA Synthesis Kit with dsDNase (Thermo Fisher Scientific, Vilnius, Lithuania) with oligo(dT) as primers. Real-Time PCR was carried out using the 5 × HOT FIREPol EvaGreen qPCR Mix Plus, no ROX (Solis BioDyne, Tartu, Estonia) and Rotor-Gene 6000q series (Corbett Life Science, Mortlake, Australia) according to the manufacturer’s protocol. The *ADP-ribosylation factor* (*AFR*) and *Glyceraldehyde-3-phosphate dehydrogenase* (*GAPDH*) were used as internal controls. The gene-specific primers used for the Real-Time PCR are listed in Table S1. The relative expression of the genes was calculated from the number of copies of the investigated genes and the geometric mean of the reference genes, i.e., *ARF* and *GAPDH,* which were determined using the Rotor-Gene 6000 series software 1.7 (Corbett Life Science, Chadstone, Australia).

### 4.6. Data Analysis

Statistical analyses (Student’s *t*-test) were performed using the Microsoft Excel 2016 (Microsoft Office, Warszawa, Poland) and the statistical software for the Excel, i.e., XLSTST (Addinsoft, Paris, France). Two-way ANOVA were performed using the Statistica 13.0 (StatSoft, Kraków, Poland). All of the phenotypical traits are shown as the results for six biological replicates. Three biological replicates were performed for each gene and three technical repeats for each sample. The mean values with a standard deviation were presented. Graphs were generated using the Microsoft Excel 2016 and the Microsoft PowerPoint 2016 (Microsoft Office, Warszawa, Poland). Results of the experiments are presented in Table S2.

## 5. Conclusions

The BZR1 in plants was detected in three forms with different MWs corresponding to the phosphorylation level of serine/threonine residues. Additionally, the activity of *Hv*BZR1 was organ-specific. Phosphorylated forms of the *Hv*BZR1 with the cytoplasmic localization were characteristic for the shoots. In contrast, *Hv*BZR1 in roots was present as a dephosphorylated form with nuclear localization. The changes in the accumulation of dephosphorylated and phosphorylated forms of *Hv*BZR1 were observed between the Golden Promise and Haruna Nijo cultivars.

## Figures and Tables

**Table 1:** Results of two-way ANOVA for the genotype (Golden Promise and Haruna Nijo) or treatment (0.001 and 1 µM 24-EBL, 10 and 50 µM bikinin, 10 and 50 µM Brz) calculated for different growth parameters (shoot and root length, their fresh and dry weight, and related shoot-to-root ratio) and genes (*HvDWF4*, *HvBRI1*, *HvBAK1*, *HvBSU1*, *HvGSK3, HvGSK1.1*, *HvGSK1.2*, *HvGSK1.3*, *HvGSK2.1*, *HvGSK2.2*, *HvGSK3.1*, *HvGSK4.1*, *HvBZR1)*. The asterisks indicate significant dependence for *p* < 0.05 (*), *p* < 0.01 (**), and *p* < 0.001 (***).

Organ	Parameters	Genotype			Treatment		
		Mean Square	F	*p*	Mean Square	F	*p*
Shoot	length	129.407 ***	237.227	0.0000	6.445 ***	11.815	0.0000
	fresh weight	2.371 × 10^−2^ ***	129.722	0.0000	1.004 × 10^−3^ ***	5.493	0.0000
	dry weight	1.584 × 10^−4^ ***	95.728	0.0000	7.180 × 10^−6^ ***	4.339	0.0002
	RWC	4.747 **	10.340	0.0018	2.084 ***	4.539	0.0001
	*HvDWF4*	3.937 × 10^−7^ **	8.924	0.0046	6.764 × 10^−7^ ***	15.330	0.0000
	*HvBRI1*	1.912 × 10^−3^	2.345	0.1328	3.513 × 10^−3^ ***	4.308	0.0007
	*HvBAK1*	4.246 × 10^−3^ ***	572.952	0.0000	2.322 × 10^−5^ **	3.133	0.0069
	*HvBSU1*	2.924 × 10^−3^ ***	47.809	0.0000	1.269 × 10^−4^	2.075	0.0591
	*HvGSK1.1*	5.645 × 10^−3^ *	4.271	0.0447	3.885 × 10^−3^ *	2.939	0.0101
	*HvGSK1.2*	5.311 × 10^−2^ ***	30.925	0.0000	1.850 × 10^−3^	1.077	0.3963
	*HvGSK1.3*	0.101 ***	203.986	0.0000	1.273 × 10^−3^ *	2.560	0.0219
	*HvGSK2.1*	0.131 ***	79.846	0.0000	3.586 × 10^−3^ *	2.180	0.0477
	*HvGSK2.2*	1.915 × 10^−2^ ***	99.207	0.0000	7.749 × 10^−4^ **	4.015	0.0012
	*HvGSK3.1*	4.833 × 10^−3^ ***	39.596	0.0000	2.983 × 10^−4^ *	2.444	0.0278
	*HvGSK4.1*	1.091 × 10^−2^ ***	71.189	0.0000	4.283 × 10^−4^ *	2.794	0.0136
	*HvBZR1*	6.562 × 10^−6^ ***	68.418	0.0000	1.873 × 10^−7^	1.953	0.0756
Root	length	4.400	2.093	0.1512	49.539 ***	23.562	0.0000
	fresh weight	7.634 × 10^−5^	0.438	0.5096	4.306 × 10^−3^ ***	24.715	0.0000
	dry weight	9.600 × 10^−6^ **	7.274	0.0082	1.508 × 10^−5^ ***	11.428	0.0000
	RWC	13.306 **	7.328	0.0080	20.335 ***	11.199	0.0000
	*HvDWF4*	0.226	1.008	0.3209	0.218	0.974	0.4688
	*HvBRI1*	3.745 × 10^−3^	0.222	0.6393	2.975 × 10^−2^	1.760	0.1121
	*HvBAK1*	4.879 × 10^−2^	3.178	0.0817	1.548 × 10^−2^	1.008	0.4442
	*HvBSU1*	1.788 × 10^−2^	1.430	0.2384	1.163 × 10^−2^	0.930	0.5017
	*HvGSK1.1*	5.917 × 10^−2^ *	4.195	0.0467	1.689 × 10^−2^	1.193	0.3233
	*HvGSK1.2*	3.394 × 10^−2^	2.637	0.1117	1.371 × 10^−2^	1.065	0.4051
	*HvGSK1.3*	8.160 × 10^−3^	0.539	0.4669	1.589 × 10^−2^	1.049	0.4156
	*HvGSK2.1*	0.111 **	9.515	0.0036	9.758 × 10^−3^	0.838	0.5747
	*HvGSK2.2*	4.686 × 10^−2^	2.950	0.0931	1.463 × 10^−2^	0.921	0.5087
	*HvGSK3.1*	3.283 × 10^−2^	1.368	0.2485	2.429 × 10^−2^	1.012	0.4412
	*HvGSK4.1*	2.724 × 10^−2^	2.160	0.1489	1.036 × 10^−2^	0.821	0.5882
	*HvBZR1*	9.796 × 10^−4^	0.519	0.4751	1.752 × 10^−2^	0.928	0.5031
Shoot-to-root ratio	by length	0.677 ***	31.750	0.0000	0.621 ***	29.098	0.0000
	by fresh weight	4.545 ***	180.744	0.0000	1.361 ***	54.135	0.0000
	by dry weight	6.411 ***	115.179	0.0000	1.111 ***	19.953	0.0000

## Data Availability

Data are contained within the article or Supplementary Materials.

## Supplementary Materials

The following are available online at https://www.mdpi.com/article/10.3390/ijms222413572/s1.

## References

[1] Bajguz A. (2007). Metabolism of brassinosteroids in plants. Plant Physiol. Biochem..

[2] Shimada Y., Goda H., Nakamura A., Takatsuto S., Fujioka S., Yoshida S. (2003). Organ-specific expression of brassinosteroid-biosynthetic genes and distribution of endogenous brassinosteroids in Arabidopsis. Plant Physiol..

[3] Zhang C., Bai M.-Y., Chong K. (2014). Brassinosteroid-mediated regulation of agronomic traits in rice. Plant Cell Rep..

[4] Feng Z., Wu C., Wang C., Roh J., Zhang L., Chen J., Zhang S., Zhang H., Yang C., Hu J. (2016). SLG controls grain size and leaf angle by modulating brassinosteroid homeostasis in rice. J. Exp. Bot..

[5] Xiao Y., Zhang G., Liu D., Niu M., Tong H., Chu C. (2020). GSK2 stabilizes OFP3 to suppress brassinosteroid responses in rice. Plant J..

[6] Wang Z.-Y., Bai M.-Y., Oh E., Zhu J.-Y. (2012). Brassinosteroid signaling network and regulation of photomorphogenesis. Annu. Rev. Genet..

[7] Duan P., Ni S., Wang J., Zhang B., Xu R., Wang Y., Chen H., Zhu X., Li Y. (2015). Regulation of OsGRF4 by OsmiR396 controls grain size and yield in rice. Nat. Plants.

[8] Duan P., Rao Y., Zeng D., Yang Y., Xu R., Zhang B., Dong G., Qian Q., Li Y. (2014). SMALL GRAIN 1, which encodes a mitogen-activated protein kinase kinase 4, influences grain size in rice. Plant J..

[9] Li Y., Fan C., Xing Y., Jiang Y., Luo L., Sun L., Shao D., Xu C., Li X., Xiao J. (2011). Natural variation in GS5 plays an important role in regulating grain size and yield in rice. Nat. Genet..

[10] Wang S., Wu K., Yuan Q., Liu X., Liu Z., Lin X., Zeng R., Zhu H., Dong G., Qian Q. (2012). Control of grain size, shape and quality by OsSPL16 in rice. Nat. Genet..

[11] Nakagawa H., Tanaka A., Tanabata T., Ohtake M., Fujioka S., Nakamura H., Ichikawa H., Mori M. (2012). Short grain1 decreases organ elongation and brassinosteroid response in rice. Plant Physiol..

[12] Corvalan C., An G., Choe S. (2021). The Rice propiconazole resistant 1-D mutant, with activated expression of a DPb transcription factor gene, exhibits increased seed yields. bioRxiv.

[13] Bittner T., Campagne S., Neuhaus G., Rensing S.A., Fischer-Iglesias C. (2013). Identification and characterization of two wheat Glycogen Synthase Kinase 3/SHAGGY-like kinases. BMC Plant Biol..

[14] Hartwig T., Corvalan C., Best N.B., Budka J.S., Zhu J.Y., Choe S., Schulz B. (2012). Propiconazole is a specific and accessible brassinosteroid (BR) biosynthesis inhibitor for Arabidopsis and maize. PLoS ONE.

[15] Gruszka D. (2020). Exploring the Brassinosteroid Signaling in Monocots Reveals Novel Components of the Pathway and Implications for Plant Breeding. Int. J. Mol. Sci..

[16] Cui X.-Y., Gao Y., Guo J., Yu T.-F., Zheng W.-J., Liu Y.-W., Chen J., Xu Z.-S., Ma Y.-Z. (2019). BES/BZR Transcription Factor TaBZR2 Positively Regulates Drought Responses by Activation of TaGST1. Plant Physiol..

[17] Corvalan C., Choe S. (2017). Identification of brassinosteroid genes in Brachypodium distachyon. BMC Plant Biol..

[18] Groszyk J., Szechyńska-Hebda M. (2021). Effects of 24-Epibrassinolide, Bikinin, and Brassinazole on Barley Growth under Salinity Stress Are Genotype- and Dose-Dependent. Agronomy.

[19] Castorina G., Consonni G. (2020). The Role of Brassinosteroids in Controlling Plant Height in Poaceae: A Genetic Perspective. Int. J. Mol. Sci..

[20] Nolan T.M., Vukašinović N., Liu D., Russinova E., Yin Y. (2020). Brassinosteroids: Multidimensional regulators of plant growth, development, and stress responses. Plant Cell.

[21] Oh M.-H., Honey S.H., Tax F.E. (2020). The Control of Cell Expansion, Cell Division, and Vascular Development by Brassinosteroids: A Historical Perspective. Int. J. Mol. Sci..

[22] Mao J., Li J. (2020). Regulation of Three Key Kinases of Brassinosteroid Signaling Pathway. Int. J. Mol. Sci..

[23] He J.-X., Gendron J.M., Sun Y., Gampala S.S., Gendron N., Sun C.Q., Wang Z.-Y. (2005). BZR1 is a transcriptional repressor with dual roles in brassinosteroid homeostasis and growth responses. Science.

[24] Yin Y., Vafeados D., Tao Y., Yoshida S., Asami T., Chory J. (2005). A new class of transcription factors mediates brassinosteroid-regulated gene expression in Arabidopsis. Cell.

[25] Goda H., Shimada Y., Asami T., Fujioka S., Yoshida S. (2002). Microarray analysis of brassinosteroid-regulated genes in Arabidopsis. Plant Physiol..

[26] Bai M.Y., Zhang L.Y., Gampala S.S., Zhu S.W., Song W.Y., Chong K., Wang Z.Y. (2007). Functions of OsBZR1 and 14-3-3 proteins in brassinosteroid signaling in rice. Proc. Natl. Acad. Sci. USA.

[27] He Y., Hong G., Zhang H., Tan X., Li L., Kong Y., Sang T., Xie K., Wei J., Li J. (2020). The OsGSK2 kinase integrates brassinosteroid and jasmonic acid signaling by interacting with OsJAZ4. Plant Cell.

[28] Hughes P.W. (2020). OsGSK2 Integrates Jasmonic Acid and Brassinosteroid Signaling in Rice. Am. Soc. Plant. Biol..

[29] Xiao Y., Liu D., Zhang G., Tong H., Chu C. (2017). Brassinosteroids regulate OFP1, a DLT interacting protein, to modulate plant architecture and grain morphology in rice. Front. Plant Sci..

[30] Tong H., Jin Y., Liu W., Li F., Fang J., Yin Y., Qian Q., Zhu L., Chu C. (2009). DWARF AND LOW-TILLERING, a new member of the GRAS family, plays positive roles in brassinosteroid signaling in rice. Plant J..

[31] Tong H., Liu L., Jin Y., Du L., Yin Y., Qian Q., Zhu L., Chu C. (2012). DWARF AND LOW-TILLERING acts as a direct downstream target of a GSK3/SHAGGY-like kinase to mediate brassinosteroid responses in rice. Plant Cell.

[32] Tong H., Chu C. (2014). Roles of DLT in fine modulation on brassinosteroid response in rice. Plant Signal. Behav..

[33] Zhang C., Xu Y., Guo S., Zhu J., Huan Q., Liu H., Wang L., Luo G., Wang X., Chong K. (2012). Dynamics of brassinosteroid response modulated by negative regulator LIC in rice. PLoS Genet..

[34] Qiao S., Sun S., Wang L., Wu Z., Li C., Li X., Wang T., Leng L., Tian W., Lu T. (2017). The RLA1/SMOS1 transcription factor functions with OsBZR1 to regulate brassinosteroid signaling and rice architecture. Plant Cell.

[35] Gruszka D., Janeczko A., Dziurka M., Pociecha E., Oklestkova J., Szarejko I. (2016). Barley Brassinosteroid Mutants Provide an Insight into Phytohormonal Homeostasis in Plant Reaction to Drought Stress. Front Plant Sci..

[36] Oh E., Zhu J.Y., Bai M.Y., Arenhart R.A., Sun Y., Wang Z.Y. (2014). Cell elongation is regulated through a central circuit of interacting transcription factors in the Arabidopsis hypocotyl. eLife.

[37] Wang L., Wang Z., Xu Y., Joo S.H., Kim S.K., Xue Z., Xu Z., Wang Z., Chong K. (2009). OsGSR1 is involved in crosstalk between gibberellins and brassinosteroids in rice. Plant J..

[38] Yang X., Bai Y., Shang J., Xin R., Tang W. (2016). The antagonistic regulation of abscisic acid-inhibited root growth by brassinosteroids is partially mediated via direct suppression of ABSCISIC ACID INSENSITIVE 5 expression by BRASSINAZOLE RESISTANT 1. Plant Cell Environ..

[39] Sahni S., Prasad B.D., Liu Q., Grbic V., Sharpe A., Singh S.P., Krishna P. (2016). Overexpression of the brassinosteroid biosynthetic gene DWF4 in Brassica napus simultaneously increases seed yield and stress tolerance. Sci. Rep..

[40] Kim T.W., Guan S., Sun Y., Deng Z., Tang W., Shang J.X., Sun Y., Burlingame A.L., Wang Z.Y. (2009). Brassinosteroid signal transduction from cell-surface receptor kinases to nuclear transcription factors. Nat. Cell Biol..

[41] Wang Z.-Y., Nakano T., Gendron J., He J., Chen M., Vafeados D., Yang Y., Fujioka S., Yoshida S., Asami T. (2002). Nuclear-localized BZR1 mediates brassinosteroid-induced growth and feedback suppression of brassinosteroid biosynthesis. Dev. Cell.

[42] Gruszka D., Szarejko I., Maluszynski M. (2011). New allele of HvBRI1 gene encoding brassinosteroid receptor in barley. J. Appl. Genet..

[43] Gruszka D., Szarejko I., Maluszynski M. (2011). Identification of barley DWARF gene involved in brassinosteroid synthesis. Plant Growth Regul..

[44] Saidi Y., Hearn T.J., Coates J.C. (2012). Function and evolution of ‘green’ GSK3/Shaggy-like kinases. Trends Plant Sci..

[45] Yoo M.-J., Albert V.A., Soltis P.S., Soltis D.E. (2006). Phylogenetic diversification of glycogen synthase kinase 3/SHAGGY-like kinase genes in plants. BMC Plant Biol..

[46] Groszyk J., Yanushevska Y., Zielezinski A., Nadolska-Orczyk A., Karlowski W.M., Orczyk W. (2018). Annotation and profiling of barley GLYCOGEN SYNTHASE3/Shaggy-like genes indicated shift in organ-preferential expression. PLoS ONE.

[47] Tanveer M., Shahzad B., Sharma A., Biju S., Bhardwaj R. (2018). 24-Epibrassinolide; an active brassinolide and its role in salt stress tolerance in plants: A review. Plant Physiol. Biochem..

[48] De Rybel B., Audenaert D., Vert G., Rozhon W., Mayerhofer J., Peelman F., Coutuer S., Denayer T., Jansen L., Nguyen L. (2009). Chemical inhibition of a subset of Arabidopsis thaliana GSK3-like kinases activates brassinosteroid signaling. Chem. Biol..

[49] Bajguz A., Chmur M., Gruszka D. (2020). Comprehensive overview of the brassinosteroid biosynthesis pathways: Substrates, products, inhibitors, and connections. Front. Plant Sci..

[50] Min H.J., Cui L.H., Oh T.R., Kim J.H., Kim T.W., Kim W.T. (2019). OsBZR 1 turnover mediated by Os SK 22-regulated U-box E3 ligase Os PUB 24 in rice BR response. Plant J..

[51] Gao X., Zhang J.-Q., Zhang X., Zhou J., Jiang Z., Huang P., Tang Z., Bao Y., Cheng J., Tang H. (2019). Rice qGL3/OsPPKL1 functions with the GSK3/SHAGGY-like kinase OsGSK3 to modulate brassinosteroid signaling. Plant Cell.

[52] Zhu X., Liang W., Cui X., Chen M., Yin C., Luo Z., Zhu J., Lucas W.J., Wang Z., Zhang D. (2015). Brassinosteroids promote development of rice pollen grains and seeds by triggering expression of Carbon Starved Anther, a MYB domain protein. Plant J..

[53] Fang Z., Ji Y., Hu J., Guo R., Sun S., Wang X. (2020). Strigolactones and brassinosteroids antagonistically regulate the stability of the D53–OsBZR1 complex to determine FC1 expression in rice tillering. Mol. Plant.

[54] (2021). UniProt: The universal protein knowledgebase in 2021. Nucleic Acids Res..

[55] O’Leary N.A., Wright M.W., Brister J.R., Ciufo S., Haddad D., McVeigh R., Rajput B., Robbertse B., Smith-White B., Ako-Adjei D. (2016). Reference sequence (RefSeq) database at NCBI: Current status, taxonomic expansion, and functional annotation. Nucleic Acids Res..

[56] Howe K.L., Contreras-Moreira B., De Silva N., Maslen G., Akanni W., Allen J., Alvarez-Jarreta J., Barba M., Bolser D.M., Cambell L. (2020). Ensembl Genomes 2020—enabling non-vertebrate genomic research. Nucleic Acids Res..

[57] Kawahara Y., de la Bastide M., Hamilton J.P., Kanamori H., McCombie W.R., Ouyang S., Schwartz D.C., Tanaka T., Wu J., Zhou S. (2013). Improvement of the Oryza sativa Nipponbare reference genome using next generation sequence and optical map data. Rice.

[58] Wang H., Jiao X., Kong X., Liu Y., Chen X., Fang R., Yan Y. (2020). The histone deacetylase HDA703 interacts with OsBZR1 to regulate rice brassinosteroid signaling, growth and heading date through repression of Ghd7 expression. Plant J..

[59] Jeong Y.J., Corvalán C., Kwon S.I., Choe S. (2015). Analysis of anti-BZR1 antibody reveals the roles BES1 in maintaining the BZR1 levels in Arabidopsis. J. Plant Biol..

[60] Zhao M., Tang S., Zhang H., He M., Liu J., Zhi H., Sui Y., Liu X., Jia G., Zhao Z. (2020). DROOPY LEAF1 controls leaf architecture by orchestrating early brassinosteroid signaling. Proc. Natl. Acad. Sci. USA.

[61] Kim E.-J., Lee S.-H., Park C.-H., Kim S.-H., Hsu C.-C., Xu S., Wang Z.-Y., Kim S.-K., Kim T.-W. (2019). Plant U-box40 mediates degradation of the brassinosteroid-responsive transcription factor BZR1 in Arabidopsis roots. Plant Cell.

[62] Yang M., Wang X. (2017). Multiple ways of BES1/BZR1 degradation to decode distinct developmental and environmental cues in plants. Mol. Plant.

[63] Yang M., Li C., Cai Z., Hu Y., Nolan T., Yu F., Yin Y., Xie Q., Tang G., Wang X. (2017). SINAT E3 ligases control the light-mediated stability of the brassinosteroid-activated transcription factor BES1 in Arabidopsis. Dev. Cell.

[64] Rozhon W., Wang W., Berthiller F., Mayerhofer J., Chen T., Petutschnig E., Sieberer T., Poppenberger B., Jonak C. (2014). Bikinin-like inhibitors targeting GSK3/Shaggy-like kinases: Characterisation of novel compounds and elucidation of their catabolism in planta. BMC Plant Biol..

[65] Liu J., Chen J., Zheng X., Wu F., Lin Q., Heng Y., Tian P., Cheng Z., Yu X., Zhou K. (2017). GW5 acts in the brassinosteroid signalling pathway to regulate grain width and weight in rice. Nat. Plants.

[66] Vukašinović N., Wang Y., Vanhoutte I., Fendrych M., Guo B., Kvasnica M., Jiroutová P., Oklestkova J., Strnad M., Russinova E. (2021). Local brassinosteroid biosynthesis enables optimal root growth. Nat. Plants.

[67] Lee H.-S., Kim Y., Pham G., Kim J.W., Song J.-H., Lee Y., Hwang Y.-S., Roux S.J., Kim S.-H. (2015). Brassinazole resistant 1 (BZR1)-dependent brassinosteroid signalling pathway leads to ectopic activation of quiescent cell division and suppresses columella stem cell differentiation. J. Exp. Bot..

[68] Chaiwanon J., Wang Z.Y. (2015). Spatiotemporal brassinosteroid signaling and antagonism with auxin pattern stem cell dynamics in Arabidopsis roots. Curr. Biol..

[69] Vilarrasa-Blasi J., González-García M.-P., Frigola D., Fàbregas N., Alexiou K.G., López-Bigas N., Rivas S., Jauneau A., Lohmann J.U., Benfey P.N. (2014). Regulation of plant stem cell quiescence by a brassinosteroid signaling module. Dev. Cell.

[70] Kim S.-K., Chang S.C., Lee E.J., Chung W.-S., Kim Y.-S., Hwang S., Lee J.S. (2000). Involvement of brassinosteroids in the gravitropic response of primary root of maize. Plant Physiol..

[71] Kim T.W., Lee S.M., Joo S.H., Yun H.S., Lee Y., Kaufman P.B., Kirakosyan A., Kim S.H., Nam K.H., Lee J.S. (2007). Elongation and gravitropic responses of Arabidopsis roots are regulated by brassinolide and IAA. Plant Cell Environ..

[72] Li L., Xu J., Xu Z.-H., Xue H.-W. (2005). Brassinosteroids stimulate plant tropisms through modulation of polar auxin transport in Brassica and Arabidopsis. Plant Cell.

[73] Inahashi H., Shelley I.J., Yamauchi T., Nishiuchi S., Takahashi-Nosaka M., Matsunami M., Ogawa A., Noda Y., Inukai Y. (2018). OsPIN2, which encodes a member of the auxin efflux carrier proteins, is involved in root elongation growth and lateral root formation patterns via the regulation of auxin distribution in rice. Physiol. Plant..

[74] Roddick J.G. (1994). Comparative root growth inhibitory activity of four brassinosteroids. Phytochemistry.

[75] Kutschera U., Wang Z.-Y. (2012). Brassinosteroid action in flowering plants: A Darwinian perspective. J. Exp. Bot..

