# Supplementary material for: Brassinazole Resistant 1 Activity Is Organ-Specific and Genotype-Dependent in Barley Seedlings
Source: Int J Mol Sci. 2021 Dec 17;22(24):13572. doi: 10.3390/ijms222413572 (PMC8706524; doi:10.3390/ijms222413572)
Supplement: Supplementary file 1 [file ijms-22-13572-s001.zip › Supplementary Material - Figures S1-3, Table S1.pdf]

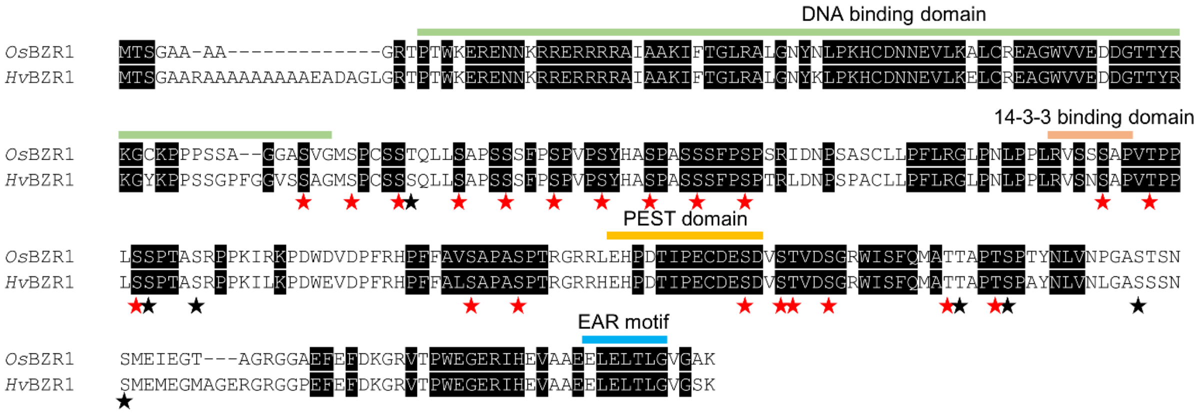

Figure S1. The amino acid sequence of *Hv*BZR1 (BAJ85589.1) and *Os*BZR1 (Q7XI96) contains DNA binging domain (green line), 14-3-3 binging domain (brown line), PEST domain (yellow line), and EAR motif (blue line). The red asterisk indicates serine and threonine residues which are phosphorylated in *Arabidopsis thaliana* and *Oryza sativa* (21 sites), the black asterisk indicates other serine and threonine residues which are phosphorylated in *O. sativa* (7 sites). The black background marks regions that are similar between *A. thaliana*, *O. sativa*, and *H. vulgare*.

```
OsBZR1          ATGACGTCCGGGGCGGCG---GCGGCG---------------------------------  18
HvBZR1          ATGACGTCGGGGGCTGCCCGGGCGGCGGCGGCGGCGGCGGCTGCGGAGGCGGACGCC  60
                ******** ****  **    ******

OsBZR1          ------GGGAGGACGCCGACGTGGAAGGAGAGGGAGAACAACAAGAGGCGGGAGCGGCGG  78
HvBZR1          GGGCTGGGGCGGACGCCGACGTGGAAGGAGCGGGAGAACAACAAGCGGCGCGAGCGGCGG  120
                      *** ******************** ************** **** *********

OsBZR1          CGGCGTGCCATCGCCGCCAAGATCTTCACGGGGCTCCGGGCGCTCGGGAACTACAACCTC  138
HvBZR1          CGCCGGGCCATCGCCGCCAAGATCTTCACCGGCCTCCGCGCGCTCGGCAACTACAAGCTC  180
                ** ** *********************** ** ***** ******** ******** ***

OsBZR1          CCCAAGCACTGCGACAACAACGAGGTGCTCAAGGCGCTCTGCCGCGAGGCCGGCTGGGTT  198
HvBZR1          CCCAAGCACTGCGACAACAACGAGGTGCTCAAGGAGCTCTGCCGCGAGGCCGGATGGGTA  240
                ********************************** ****************** *****

OsBZR1          GTCGAGGACGACGGCACCACCTACCGCAAGGGATGTAAGCCGCCGCCATCGT------CG  252
HvBZR1          GTGGAGGACGACGGCACCACCTACCGCAAGGGATACAAGCCGCCGTCGTCCGGGCCGTTC  300
                ** *******************************  ********* * **

OsBZR1          GCTGGGGGAGCGTCGGTGGGGATGAGCCCCTGCTCGTCAACGCAGCTGCTGAGCGCGCCG  312
HvBZR1          GGTGGGGTCTCGTCGGCGGGCATGAGCCCCTGCTCGTCCTCGCAGCTGCTCAGCGCGCCG  360
                * *****   ****** *** *****************  ********** *********

OsBZR1          TCGTCGTCGTTCCCGAGCCCGGTGCCGTCGTACCACGCGAGCCCGGCGTCGTCGAGCTTC  372
HvBZR1          TCGTCGTCGTTCCCGAGCCCGGTGCCTTCCTACCACGCCAGCCCGGCCTCGTCGAGCTTC  420
                ************************** ** ******** ******** ************

OsBZR1          CCGAGCCCCAGCCGGATCGACAACCCGAGCGCCTCCTGCCTCCTCCCGTTCCTCCGGGGG  432
HvBZR1          CCGAGCCCCACGCGCCTCGACAACCCGAGCCCCGCCTGCCTCCTCCCGTTCCTCCGTGGC  480
                **********  **  ************** ** ********************** **

OsBZR1          CTCCCCAACCTCCCGCCGCTCCGCGTCTCCAGCAGCGCGCCCGTCACGCCGCCGCTCTCG  492
HvBZR1          CTCCCCAACCTGCCCCCGCTCCGGGTCTCCAACAGCGCGCCAGTGACGCCGCCGCTCTCG  540
                *********** ** ******** ******* ********* ** ***************

OsBZR1          TCGCCGACGGCGTCGCGGCCGCCCAAGATCAGGAAGCCGGACTGGGACGTCGACCCGTTC  552
HvBZR1          TCGCCGACGGCGTCGCGCCCGCCCAAGATCCTGAAGCCCGACTGGGAGGTCGACCCGTTC  600
                ***************** ************  ****** ******** ************

OsBZR1          CGGCACCCCTTCTTCGCGGTCTCCGCGCCGGCGAGCCCCACCCGCGGCCGCCGCCTCGAG  612
HvBZR1          CGGCACCCGTTCTTCGCCCTCTCCGCGCCGGCGAGCCCCACCCGTGGCCGCCGGCACGAG  660
                ******** ********  ************************* ******** * ****

OsBZR1          CACCCGGACACGATACCGGAGTGCGACGAGTCCGACGTCTCCACGGTGGACTCCGGCCGG  672
HvBZR1          CATCCGGACACGATACCGGAGTGCGACGAGTCGGACGTCTCCACGGTGGACTCTGGCCGG  720
                ** ***************************** ******************** ******

OsBZR1          TGGATCAGCTTCCAGATGGCCACGACGGCGCCGACGTCGCCCACCTACAACCTCGTCAAC  732
HvBZR1          TGGATCAGCTTCCAGATGGCCACGACGGCGCCGACGTCCCCCGCGTACAACCTCGTCAAC  780
                ************************************** *** * ***************

OsBZR1          CCGGGCGCCTCCACCTCCAACTCCATGGAGATAGAAGGGACGG---------CCGGCCGA  783
HvBZR1          CTAGGCGCCTCCAGCTCAAACTCCATGGAGATGGAGGGAATGGCGGGGGAGAGGGGCCGA  840
                *  ********** *** ************** ** ** * **           ******

OsBZR1          GGCGGCGCGGAGTTCGAGTTCGACAAGGGGAGGGTGACGCCATGGGAGGGCGAGAGGATC  843
HvBZR1          GGCGGCCCGGAATTCGAGTTCGACAAGGGGAGGGTGACGCCGTGGGAAGGGGAAAGGATC  900
                ****** **** ***************************** ***** ** ** ******

OsBZR1          CACGAGGTCGCCGCCGAGGAGCTCGAGCTCACGCTCGGCGTCGGCGCGAAATGA    897
HvBZR1          CATGAGGTCGCCGCCGAGGAGCTTGAGCTCACGCTCGGCGTCGGCTCAAAATGA    954
                ** ******************** ********************* * ******
```

Figure S2. Alignment of the full-length coding sequences of the BZR1 gene for rice (*OsBZR1*, Os07t0580500-01) and barley (*HvBZR1*, AK354370). Asterisk indicates the same nucleotides between plant species.

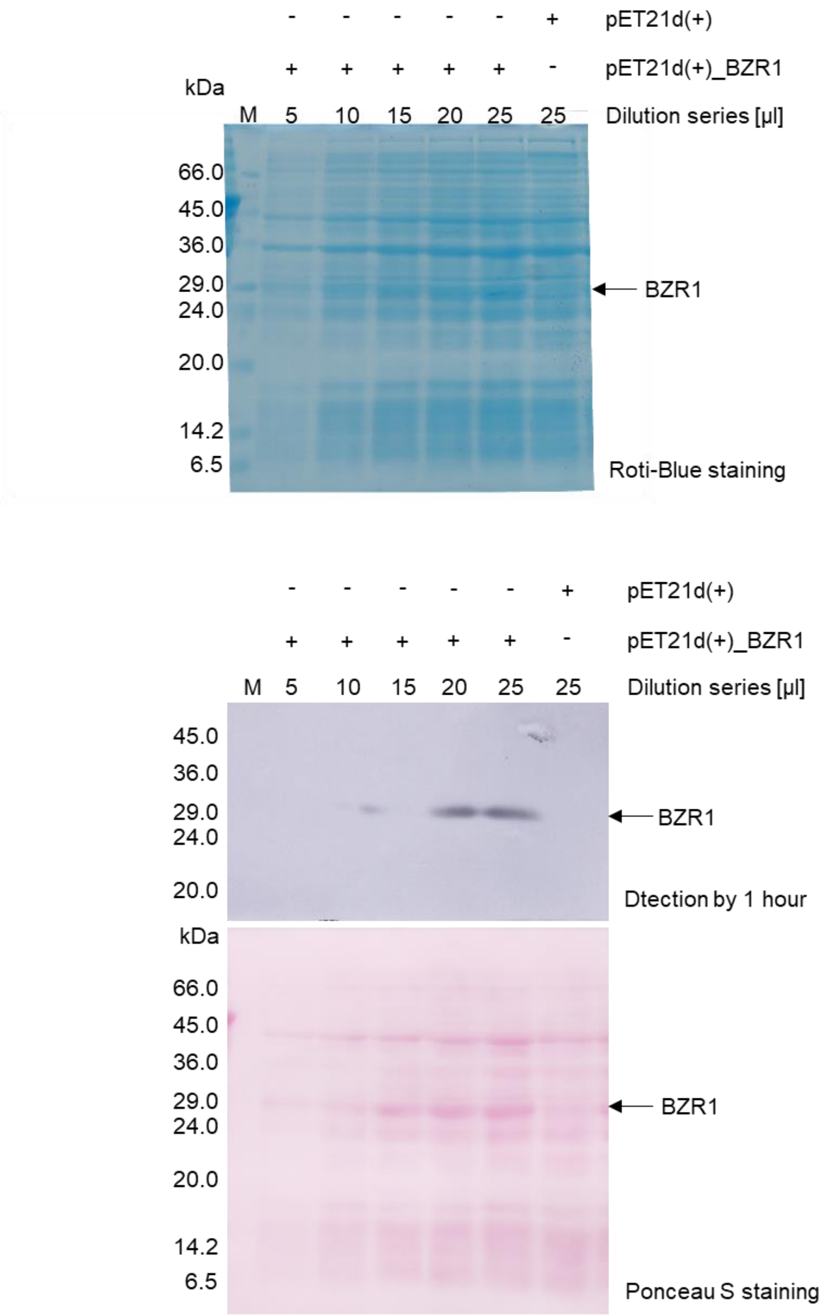

Figure S3. Results of Roti-Blue (Roth) staining of polyacrylamide gel after SDS-PAGE. Results of dilution series *E. coli* BL21(DE3) cell lysate with recombinant protein of *Hv*BZR1 (pET21d(+)_BZR1) compared to control (pET21d(+)_empty). M, Sigma Marker wide range, Molecular Weight 6.5-200.0 in kDa (Sigma); red arrow, amplicon about appropriate length.

Table S1. List of primers used in Real-Time PCR.

Gen Name	Orientation	5′–3′ Sequence	Data Base ID	Literature
HvDWF4	Forward	CATCTCCTTGCCCATCAATC	NCBI GenBank: DQ832258	Gruszka et al.2011
	Reverse	ATCGAAGTCGTGGACATGGT		
HvBAK1	Forward	TGCTGCCTTGCTATTTGCTA	NCBI GenBank: EF216861	Gruszka et al.2011
	Reverse	CTTGCCAAATCCACCTCTTC		
HvBRI1	Forward	GTCTCTGTCAAGCATTCCCG	NCBI GenBank: AB088206	not published
	Reverse	AGCTTCCCAAGCCACGA		
HvBSU1	Forward	TGGCATCGCGTGAATAGGTT	NCBI GenBank: AK375108.1	not published
	Reverse	AACAACTGAGCCTGCTTCCA		
HvGSK1.1	Forward	CCCTTCTTTGATGAGCTTCG	Ensemble Plants: HORVU3Hr1G034440.2	Groszyk et. al.2018
	Reverse	CAGGGGAAATGCTCACTTGT		
HvGSK1.2	Forward	TCTGGGCACACCTACAAGGG	Ensemble Plants: HORVU5Hr1G117030.1	Groszyk et. al.2018
	Reverse	TGGAGACCAGGTCCACTGCT		
HvGSK1.3	Forward	ACGAGATGGGCAATATGAG	Ensemble Plants: HORVU1Hr1G016490.9	Groszyk et. al.2018
	Reverse	GTTCCAAATGACCCATGACC		
HvGSK2.1	Forward	AGTGCTTGGAGACTGGAGAGAC	Ensemble Plants: HORVU3Hr1G026020.1	Groszyk et. al.2018
	Reverse	GTGCTTCAGAGAGACGACATTG		
HvGSK2.2	Forward	CACCAACTCGGGAGGAAATA	Ensemble Plants: MLOC_68311.2	Groszyk et. al.2018
	Reverse	GCTCCCGTAGCTCATCAAAG		
HvGSK3.1	Forward	AAAGTGGCGTTGATCAGTTGG	Ensemble Plants: HORVU1Hr1G048580.7	Groszyk et. al.2018
	Reverse	CAGGGATGAGCTTTTATCTGAGG		
HvGSK4.1	Forward	GCGAGAAGGCAGAACCTGTT	Ensemble Plants: HORVU5Hr1G119790.18	Groszyk et. al.2018
	Reverse	TGTCACCCACCCACACAAAG		
HvBZR1	Forward	CCCGTTCTTCGCCCTCTC	NCBI GenBank: AJ508228	not published
	Reverse	ATCTGGAAGCTGATCCACCG		
HvGAPDH	Forward	TCAAGCAAGGACTGGAGAGG	NCBI GenBank: X60343	Gruszka et al.2011
	Reverse	ACACATCCACAGTGGGAACC		
HvARF	Forward	GCTCTCCAACAACATTGCCAAC	NCBI GenBank: AJ508228	Groszyk et. al.2018
	Reverse	GCTTCTGCCTGTCACATACGC		

Groszyk J, Yanushevska Y, Zielezinski A, Nadolska-Orczyk A, Karlowski WM, Orczyk W (2018) Annotation and profiling of barley GLYCOGEN SYNTHASE3/Shaggy-like genes indicated shift in organ-preferential expression. PLoS One 13(6): e0199364

Gruszka D, Szarejko I, Maluszynski M (2011) Identification of barley DWARF gene involved in brassinosteroid synthesis. Plant Growth Regulation 65(2): 343-358
